# Effects of Concentration, Salinity and Temperature on the Conformations of Zwitterionic Poly(2-Vinylpyridine‑*N*‑Oxide) Chains in Semidilute Solutions Probed by Small-Angle X‑Ray and Neutron Scattering

**DOI:** 10.1021/acs.macromol.5c02665

**Published:** 2026-03-27

**Authors:** Polyxeni P. Angelopoulou, Jong K. Keum, Pei-Chi Chen, Guang-Rong Huang, Changwoo Do, Logan T. Kearney, Jan Michael Carrillo, Yangyang Wang, Jack F. Douglas, Panagiotis Christakopoulos, Rajeev Kumar, Georgios Sakellariou, Amit K. Naskar, Kunlun Hong

**Affiliations:** † Chemical Sciences Division, 6146 Oak Ridge National Laboratory, Oak Ridge, Tennessee 37830, United States; ‡ Center for Nanophase Materials Sciences, 6146 Oak Ridge National Laboratory, Oak Ridge, Tennessee 37830, United States; § Neutron Scattering Division, 6146 Oak Ridge National Laboratory, Oak Ridge, Tennessee 37830, United States; ∥ Department of Engineering and System Science, 34881 National Tsing Hua University, Hsinchu 300044, Taiwan; ⊥ NIST Fellow Emeritus, 10833 National Institute of Standards and Technology, Gaithersburg, Maryland 20899, United States; # Department of Chemistry, National and Kapodistrian University of Athens, Athens, 15771, Greece; a Physics Division, National Center for Theoretical Sciences, Taipei 10617, Taiwan

## Abstract

Polyzwitterions, composed of repeating units with equal numbers of anionic and cationic groups, have drawn considerable interest for applications ranging from antimicrobial coatings and antifouling membranes to low-friction interfaces, energy storage media, and actuators. A characteristic feature of many polyzwitterions is the anti-polyelectrolyte effect—salt-induced chain expansion in aqueous solution—a phenomenon often implicated in their functional behavior. In this study, we investigated the conformational behavior of poly­(2-vinylpyridine-*N*-oxide) (P2VPNO) using small-angle X-ray and neutron scattering (SAXS and SANS). Small-angle X-ray scattering (SAXS) revealed that, in salt-free aqueous solution, P2VPNO adopts an expanded wormlike conformation stabilized by hydration. With increasing concentration, the chains contract due to screening of intrachain excluded-volume interactions, qualitatively consistent with de Gennes’ scaling predictions for neutral polymers. Small-angle neutron scattering (SANS) further demonstrated that P2VPNO exhibits the characteristic anti-polyelectrolyte response to added salt, as observed in many other polyzwitterions. At elevated temperatures, chain flexibility increases, leading to a shorter Kuhn length and reduced radius of gyration. Notably, however, salt-induced chain expansion persists, indicating that the anti-polyelectrolyte effect remains operative under elevated thermal conditions. These findings provide the first experimental evidence of scaling behavior in polyzwitterions, as well as the first observation of their altered anti-polyelectrolyte response at elevated temperatures, offering new insights into the solution physics of this important class of polymers.

## Graphical abstract

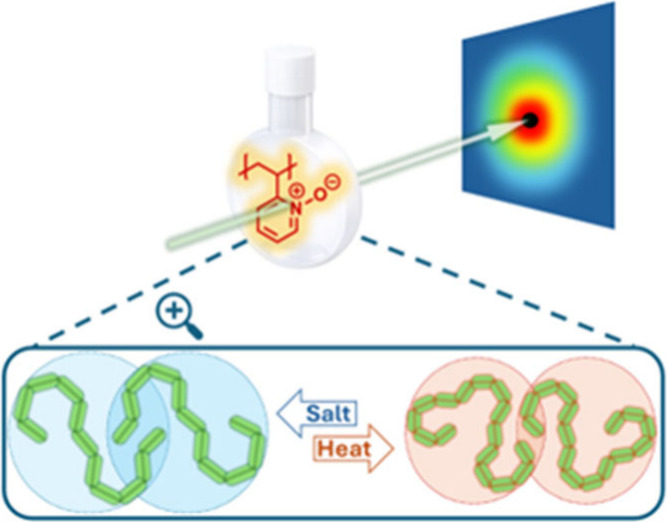

## Introduction

Polyzwitterions (PZs) are a special class of charge–containing polymers characterized by pairs of positive and negative charges within each repeating unit.^1,2^ This intrinsic charge balance, resulting in an overall zero net charge, distinguishes them from typical polyelectrolytes, i.e., polyanions and polycations with apparent non–zero net charges.^3–5^ One of the common properties of PZs is their “anti-polyelectrolyte” behavior in aqueous solutions.^6–8^ Unlike ordinary polyelectrolytes, where added salt normally induces chain collapse,^9,10^ PZs exhibit chain expansion upon the addition of salts, which defines the anti-polyelectrolyte effect.^6–8,11^ In salt-free dilute solutions, the chain conformations of PZs are mostly governed by the competition between intramolecular ‘dipole–dipole’ attractions and polymer–water interactions.^8,9,12,13^ When polymer–water interactions dominate, as in good solvents, the chains hydrate and expand. In contrast, stronger intramolecular dipole–dipole attractions, as in poor solvents, favor a compact, collapsed conformation. Beyond this solvent–quality effect, concentration can also play a central role in governing the conformations of PZs. For neutral linear polymers in good solvents, de Gennes’ scaling theory predicts that above the overlap concentration, interpenetration of neighboring chains progressively screens intrachain excluded–volume interactions, resulting in a reduced radius of gyration (*R*_*g*_).^14–16^ Similarly, PZs are often assumed to follow the same scaling behavior as neutral polymers, yet systematic studies confirming such trends remain lacking.

One of the key distinctions between PZs and neutral linear polymers is that their conformations are governed not only by solvent quality and concentration but also by the presence of added salts. Upon salt addition, ions screen dipolar interactions, weakening the intramolecular dipolar attractions and reducing the extent of intramolecular association within the chain–an effect known as the anti-polyelectrolyte effect. As a result, the chains expand, leading to an increase in *R*_*g*_, with the extent of chain expansion depending on salt concentration and residual intramolecular associations. Wang et al. used Fluorescence Correlation Spectroscopy (FCS) to study the chain conformation of zwitterionic poly­(sulfobetaine methacrylate) (PSBMA) with fluorescent repeat units in extremely dilute aqueous solutions at the single-chain level.^11^ FCS analysis revealed that adding inorganic and organic salts led to polymer chain expansion, with greater expansion observed as salt concentration increased past a certain threshold. Delgado et al. investigated the anti-polyelectrolyte effect in poly­(acrylamido sulfobetaine) (PAEDAPS) solutions using the Hofmeister series of salts and light scattering techniques.^6^ Their findings revealed that with NaCl, the polymer dissolved, expanded, and then remained unchanged as the salt concentration increased. However, with different salts such as NaBr, NaClO_4_ and NaSCN, the polymer exhibited more complex behaviors: it initially dissolved, remained nearly unchanged across certain salt concentration ranges, then expanded once specific thresholds were reached, and eventually stabilized despite further increases in salt concentration. Our recent results based on SAXS and dynamic light scattering (DLS) showed a slightly different aspect from the aforementioned results.^8^ Varying potassium bromide (KBr) concentration induced a non-monotonic change in the chain conformation of PSBMA, reflected in both *R*_*g*_ and hydrodynamic radius (*R*_*h*_). At very low salt concentrations, the polymer chains initially expanded, followed by a slight contraction as the salt concentration increased. Beyond this point, no further conformational changes were observed, indicating saturation of the ionic screening of intramolecular electrostatic interactions. It was shown that the PSBMA chains tend to have net positive charge in salt-free dilute aqueous solutions due to protonation. The chain expansion at very low salt concentrations was explained by the preferable binding of potassium ions to the sulfonate groups of PSBMA, which further increased the net positive charge along the PSBMA backbone. This enhanced like–charge repulsion among chain segments, analogous to polyelectrolyte behavior, resulted in chain expansion. As the KBr concentration increases, full electrostatic screening reduces these repulsions, leading to a decrease in *R*_*g*_. However, the chains remain more expanded than in the salt-free condition, emphasizing the role of ionic screening in governing the conformation of PZs in aqueous media—characteristic of the anti-polyelectrolyte effect. At higher salt concentrations, the lack of additional conformational change confirms the saturation of screening effects.

In contrast, it has been demonstrated that not all PZs exhibit anti-polyelectrolyte behavior. Kwei and co–workers studied poly­(4-vinylpyridine-*N*-oxide) (P4VPNO) in aqueous solutions using viscometry and light scattering, investigating the effects of P4VPNO concentration and lithium chloride (LiCl).^17^ They observed that, at dilute P4VPNO concentrations and in the absence of LiCl, the reduced viscosity (viscosity normalized by P4VPNO concentration) decreased with increasing concentration up to 0.2 g/dL and then stabilized at higher concentrations. Notably, in the presence of LiCl, the reduced viscosity decreased rather than increased compared to the salt-free condition, as typically observed in polyelectrolytes. This indicates that the anti-polyelectrolyte effect is absent with LiCl, with the underlying mechanism remaining unclarified. It has also been reported that poly­[2-(methacryloyloxy)­ethyl phosphorylcholine] (PMPC) does not exhibit anti-polyelectrolyte behavior.^18,19^ Studies have shown that the chain conformation of PMPC is already in a hydrated, pre–expanded state in salt-free aqueous solution, with minimal intramolecular dipolar associations along the backbone due to the highly polar, hydrophilic nature of phosphorylcholine group. As a result, PMPC chains do not exhibit noticeable chain expansion upon varying salinity conditions. Consequently, both P4VPNO and PMPC exhibit limited or no anti-polyelectrolyte behavior, unlike other PZs, which show significant chain expansion under saline conditions.

Poly­(2-vinylpyridine-*N*-oxide) (P2VPNO) is an emerging class of stimuli–responsive polymers characterized by distinctive dipolar character, originating from the charge separation across the N^+^–O^–^ bond within the pyridine-*N*-oxide group in the repeat units.^20^ Although the salt-dependent hydration and anti-polyelectrolyte behavior of P2VPNOs has not yet been reported, our previous work demonstrated that their dipolar character strongly influences their solution behavior in a variety of organic solvents.^21,22^ It is also well-known that dipoles can affect the stimuli–responsive properties of P2VPNOs to changes in pH.^23–25^ It has been reported that the pyridine-*N*-oxide group in P2VPNO has a dipole moment of ∼4.24 D, higher than that of the pyridine ring in its parent polymer, poly­(2-vinylpyridine) (P2VP), which has a dipole moment of ∼2.22 D.^20,26,27^ However, the dipole moment is much lower than those of more widely studied PZs like PSBMA (∼21.5 D), PMPC (∼15.4 D), and poly­(carboxybetaine methacrylate) (PCBMA) (∼14.5 D), primarily due to the shorter separation distance between the positive and negative charges.^28^ The dipole moment is proportional to the separation distance between the cation and anion, as expressed by the equation μ = *Qr*, where μ is the dipole moment, *Q* is charge magnitude, and *r* is the distance between the positive and negative charges, assuming *Q* is constant.^29^ The magnitude of the dipole moment significantly affects both intramolecular dipole–dipole interactions and hydration and thereby influencing the chain conformation. A higher dipole moment leads to stronger intramolecular dipole–dipole interactions, potentially leading to chain association and a more compact chain conformation. At the same time, it is directly proportional to polymer’s ability to form hydrogen bonds or dipolar interactions with water.^8,22,30^ When polymer–water interactions dominate over polymer–polymer interactions, the chains hydrate and expand. Conversely, when polymer–polymer interactions are stronger, the chains adopt a more compact conformation. The addition of salt to polymer solutions introduces another complexity by altering both intramolecular electrostatic interactions and hydration of polymers, thereby influencing the chain conformation.

This study first examines the concentration-dependent hydration and resulting conformational behavior of P2VPNO in salt-free aqueous solution using SAXS and then explores its anti-polyelectrolyte response to salt concentration and temperature using contrast-varied SANS. Contrast variation combined with constrained global model-fitting of multiple SANS data sets enabled detailed analysis of chain responses to ionic strength and temperature. Complementary SAXS measurements over a wide concentration range revealed distinct conformational transitions in salt-free solution and yielded key molecular parameters, including contour length and cross-sectional radius. Incorporating these SAXS-derived parameters into the subsequent SANS modeling reduced the number of free parameters and improved the robustness of the fits. By integrating SAXS and SANS, this study achieves a more quantitative structural characterization than conventional single-data set SANS refinement. The results on this previously unexplored PZ advance the fundamental understanding of PZ solution behavior and highlight the potential utility of P2VPNO in diverse applications.

## Experimental Section

### Synthesis of P2VPNO

A precursor P2VP homopolymer with the number-average molecular weight (*M*_*n*_) of 86.0 kDa, calculated degree of polymerization (DP_calc_) of 818 and dispersity (*Đ*) of 1.2 was prepared by established anionic polymerization techniques. The number-average molecular weight (*M*_*n*_) and dispersity (*Đ*) were determined by Size Exclusion Chromatography (SEC) in DMF. The precursor P2VP homopolymer was oxidized using the method of Ochiai to yield the desired P2VPNO material with a calculated number-average molecular weight (*M*_n,cal_) of 99.1 kDa.^20^ Particularly, 2.06 g of P2VP (pyridyl units 19.6 mmol) was dissolved in glacial acetic acid (15 mL) and 30% hydrogen peroxide (5.15 mL, 45.4 mmol) was added. The solution was allowed to stir at 75 °C for 24 h under inert atmosphere. The solution was then evaporated almost to dryness, neutralized with aq. NaOH, and purified by dialysis against deionized water (MWCO = 1 kDa). After freeze-drying, P2VPNO was obtained as a pale-yellow powder. The oxidation was confirmed by ATR-FTIR and ^1^H-NMR, as reported in our previous study.^21^ Following the same protocol, a commercially available P2VP (Sigma-Aldrich, analytical standard) with vendor-reported weight-average molecular weight of *M*_*w*_ = 159 kDa and *M*_*n*_ = 152 kDa (DP_calc_ = 1446) was also oxidized to P2VPNO, yielding *M*_*n,cal*_ of 175 kDa. In addition, a commercial poly­(4-vinylpyridine) (P4VP) sample (Sigma-Aldrich, *M*_*w*_ = 57.2 kDa, *M*_*n*_ = 44.0 kDa, *Đ* = 1.3, DP_calc_ = 418) was converted to poly­(4-vinylpyridine-*N*-oxide) (P4VPNO) with *M*_*n,cal*_ calculated as 50.7 kDa and included in the study. P2VPNO and P4VPNO are positional isomers, differing in the location of the *N*-oxide functionality on the pyridine ring, and serve as structurally analogous polymers for comparative analysis.

### Size Exclusion Chromatography (SEC)

SEC, EcoSEC Elite, Tosoh Bioscience LLC, was utilized for the SEC measurements of P2VP with *M*_*n*_ *=* 86.0 kDa. The instrument was equipped with an RI detector, UV detector, a Tosoh LenS3 Multi-Angle Light Scattering Detector (MALS), and two TSKgel GMHHR-M (L × I.D. 30 cm × 7.8 mm, 5 mm particle size) mixed bed SEC columns. The columns were housed in a thermostated oven at 55 °C. DMF was the eluent at a flow rate of 0.5 mL/min. For the conventional column calibration, P2VP standards in the range of peak molecular weight, *M*_*p*_ = 4,500–118,000 g/mol were used. SEC, Agilent 1260, Agilent Technologies, Inc.; was utilized for the SEC measurements of P2VP with *M*_*n*_ *=* 152 kDa and P4VP with *M*_*n*_ *=* 44.0 kDa. The instrument was equipped with an Agilent 1260 RI detector and a Wyatt MiniDawn Light Scattering Detector. In the case of P4VP (*M*_*n*_ *=* 44.0 kDa), two Polargel-M PL1117-6800 (L × I.D. 30 cm × 7.5 mm), mixed bed columns were used. The columns were housed in a thermostated oven at 55 °C. DMF was the eluent at a flow rate of 1.0 mL/min. Light scattering detector assuming 100% mass recovery (P4VP d*n*/d*c* = 0.16) was used to determine the polymer molecular weight and dispersity *Đ*. In the case of P2VP with *M*_*n*_ *=* 152 kDa, the same SEC unit was used but this time two PolyPore PL1113-6500 (L × I.D. 30 cm × 7.5 mm), mixed bed columns were used. The columns were housed in a thermostated oven at 30 °C. THF was the eluent at a flow rate of 1.0 mL/min.

### Nuclear Magnetic Resonance (NMR)

NMR spectra for P2VP and P2VPNO samples were obtained on a Bruker Avance NEO NMR console coupled to a 11.74 T actively shielded magnet (Magnex Scientific/Varian) operating at 499.7 MHz for proton. The spectra were acquired at 298 K in either 1,1,1,3,3,3-hexafluoro-2-propanol-*d*_2_ ((CF_3_)_2_CDOD or HFIP-*d*_2_) (4.52 ppm ^1^H-reference), or in 2,2,2-trifluoroethanol-1,1-*d*_2_ (CF_3_CD_2_OH or TFE*-d*_2_) (3.88 ppm ^1^H-reference). NMR spectra for P4VP and P4VPNO samples were obtained on a Bruker NEO Bay NMR spectrometer operating at 400 MHz for proton. The spectra were acquired at 298 K in chloroform-*d* (CDCl_3_) (7.28 ppm ^1^H-reference) for P4VP and in deuterated water (D_2_O) (4.70 ppm ^1^H-reference) for P4VPNO.

### SAXS Measurements

Solutions of P2VPNO with *M*_*n,cal*_ = 99.1 kDa in deuterated water (D_2_O) were prepared at concentrations of approximately 5, 10, 20, 30, 40, and 50 mg/mL for SAXS measurements. D_2_O was used instead of H_2_O for a better consistency with the solvent conditions used in the SANS measurements, where it served as the primary solvent component over H_2_O in the D_2_O/H_2_O mixtures. These solutions were labeled X05, X10, X20, X30, X40, and X50, with the calculated volume fractions provided in Table 1. Additional P2VPNO/D_2_O solutions with P2VPNO of *M*_*n,cal*_ = 175 kDa and approximate concentrations of 3, 5, 10, 20, 35, and 50 mg/mL were prepared for SAXS measurements and labeled Y03, Y05, Y10, Y20, Y35, and Y50, respectively, as listed in Table S1. Also, P4VPNO/D_2_O solutions with P4VPNO of *M*_*n,cal*_ = 50.7 kDa and approximate concentrations of 3, 5, 10, 20, and 40 mg/mL were prepared for SAXS measurements and labeled Z03, Z05, Z10, Z20, and Z40, respectively, as listed in Table S2. The prepared solutions were allowed to equilibrate overnight at room temperature to ensure complete dissolution before SAXS measurements. SAXS measurements were performed on a Xenocs Xeuss 3.0 instrument with a D2+ MetalJet X-ray source operating at 9.2 keV (Ga Kα, *λ* = 1.3414 Å). For SAXS measurements, solutions were placed in quartz capillaries with an outer diameter of ∼2.0 mm, a wall thickness of ∼0.01 mm, and a length of ∼80 mm, then sealed with wax. The capillaries were aligned perpendicular to the X-ray beam in transmission mode, and the capillary centering function was used to ensure proper automatic capillary alignment along the X-ray beam path. Scattered X-rays were detected using a Dectris Eiger 2R 4 M hybrid photon counting detector with a pixel size of 75 × 75 μm^2^. The sample-to-detector distance for SAXS was calibrated to 900 mm using a Silver Behenate (AgBeh) standard, and the exposure time was set to 3600 s.

**Table 1:** P2VPNO/D_2_O Solutions Prepared with P2VPNO of *M*_*n,cal*_ *=* 99.1 kDa at Different Volume Fractions (*φ*_*P*2*VPNO*_)

Solution ID	Volume fraction, *φ*_*P2VPNO*_
X05	0.0044
X10	0.0087
X20	0.0172
X30	0.0258
X40	0.0339
X50	0.0420

### SANS Measurements

Contrast-variation SANS measurements were conducted using P2VPNO (*M*_*n,cal*_ = 99.1 kDa) dissolved in solvent mixtures of D_2_O and H_2_O at varying weight ratios: 100:0, ∼95:∼5, ∼90:∼10, and ∼85:∼15. For salt-containing solutions, a predetermined amount of dry KBr was added to the D_2_O/H_2_O mixtures to achieve [VPNO]:[KBr] molar ratios of 1:0, 1:1, or 1:10 in the final P2VPNO/KBr/D_2_O/H_2_O solutions. The details of these solutions are listed in Table 2. The solutions were allowed to equilibrate overnight at room temperature prior to SANS measurements to ensure complete dissolution. All solutions were measured at four different temperatures (20 °C, 40 °C, 55 °C, 60 °C). SANS measurements were conducted on the Extended *Q*-Range Small-Angle Neutron Scattering (BL-6 EQ-SANS) diffractometer at the Spallation Neutron Source, Oak Ridge National Laboratory.^31,32^ Two configurations, with sample-to-detector distances of 4 and 2.5 m, were employed. Wavelength bands were defined by minimum wavelength settings of λ_min_ = 10 Å, and λ_min_ = 2.5 Å, respectively, to probe scattering wave vectors from *q* ∼ 0.006 to ∼ 0.7 Å^–1^. The choppers were operated at 60 Hz. Samples were loaded into 2-mm–thick quartz banjo cells and the measured data were corrected for detector sensitivity and background scattering of empty banjo cells, then scaled to absolute intensity units (cm^–1^) using a porous silica standard.^33,34^ SANS model fitting was performed using a publicly available NCNR SANS software package (https://www.nist.gov/ncnr/data-reduction-analysis/sans-software) based on Igor Pro software (WaveMetrics, Inc.).

**Table 2:** P2VPNO/D_2_O/H_2_O Solutions of P2VPNO (*M*_*n,cal*_ *=* 99.1 kDa) Prepared at Different Volume Fractions (φ_*P2VPNO*_), KBr Concentrations and Corresponding Neutron Scattering Length Densities (*n*SLDs) for SANS Measurements

Sample ID	Molar ratio [VPNO]:[KBr]	D_2_O [wt %]	H_2_O [wt %]	Solvent mixture *n*SLD [10^–6^ Å^–2^]	Volume fraction φ_*P2VPNO*_
N1	1:0	100	0	6.38	0.0194
N2	1:0	95	5	6.02	0.0194
N3	1:0	90	10	5.68	0.0192
N4	1:0	85	15	5.34	0.0190
N5	1:1	100	0	6.34	0.0193
N6	1:1	95	5	5.98	0.0193
N7	1:1	90	10	5.65	0.0194
N8	1:1	85	15	5.30	0.0185
N9	1:10	100	0	6.01	0.0187
N10	1:10	95	5	5.68	0.0181
N11	1:10	90	10	5.37	0.0178
N12	1:10	85	15	5.05	0.0183

## Results and Discussion

### Synthesis of P2VPNO and P4VPNO

The parent polymer P2VP with *M*_*n*_ of 86.0 kDa was prepared by anionic polymerization, the oxidation of P2VP toward P2VPNO with *M*_*n,cal*_ of 99.1 kDa was conducted using peroxyacetic acid, and the modification was confirmed by ^1^H-NMR spectroscopy in HFIP-*d*_2_, Figure 1(a). As can be seen in the ^1^H-NMR spectra, the pyridine ring protons appear at 6.6, 7.4, and 8.2 ppm. Upon quaternization, the pyridine-*N*-oxide protons appear at 7.3 and 8.1 ppm, with the peak at 6.6 ppm being fully disappeared implying the complete conversion of P2VP toward P2VPNO. The molecular weight was determined by SEC in DMF 0.05 M LiBr, using either conventional column calibration or light scattering assuming 100% mass recovery, and the molecular weight of P2VP was determined to be *M*_*n*_ = 86.0 kDa with *Đ* = 1.21, Figure 1(b). To investigate the effect of the resulting introduction of positive (N^+^) and negative (O^–^) charges—i.e., dipolar character (δ^+^ and δ^–^)—on the glass transition temperature in the solid state, we performed Differential Scanning Calorimetry (DSC) measurements on the P2VPNO and its precursor polymer, P2VP, which lacks dipole moments. The results are shown in Figure 1(c). The P2VPNO sample exhibited no observable *T*_*g*_ below its degradation point, in direct contrast to the precursor P2VP, which exhibited a *T*_*g*_ at 100 °C. This observation suggests that the introduction of dipolar character and the resulting dipole–dipole interactions between P2VPNO chain segments significantly restrict segmental mobility in the solid state. This behavior is consistent with our previous findings for other polyzwitterionic materials.^21^ Commercially available P2VP (Sigma-Aldrich) with *M*_*n*_ *=* 152 kDa and P4VP (Sigma-Aldrich) with *M*_*n*_ = 44.0 kDa were also converted to P2VPNO and P4VPNO, respectively, and the complete conversion to P2VPNO and P4VPNO was confirmed by ^1^H-NMR spectroscopy in HFIP-*d*_2_, as shown in Figure S1­(a) and S2­(a), respectively. The size exclusion chromatography (SEC) traces of both samples showing *M*_*n*_ are also shown in Figure S1­(b) and S2­(b), respectively.

**Figure 1:**
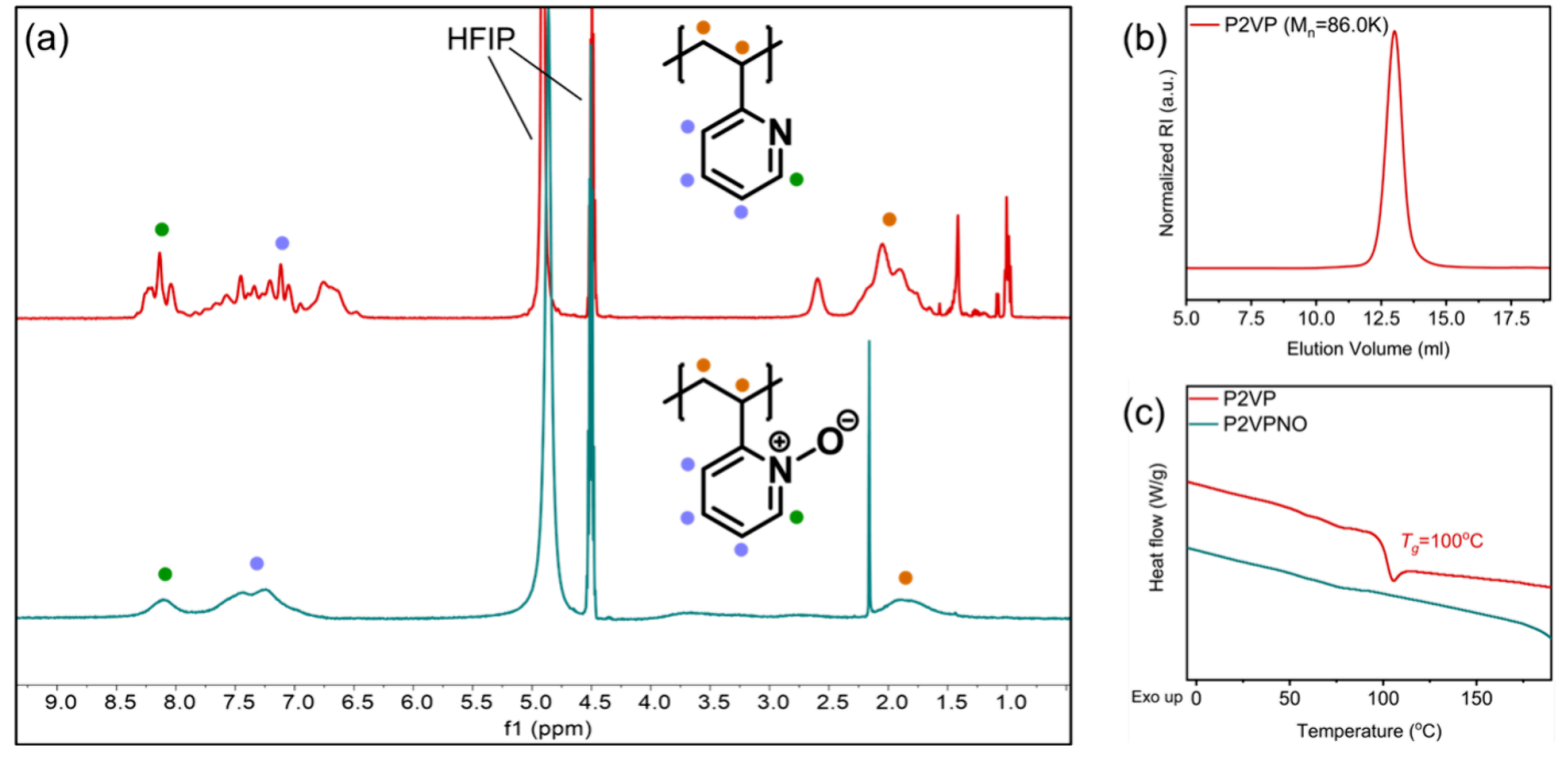
(a) ^1^H NMR in HFIP-*d*_2_ of P2VP (red) and P2VPNO (blue-green). (b) SEC trace of parent P2VP (*M*_*n*_ = 86.0 kDa) before oxidation toward P2VPNO (*M*_*n,cal*_ = 99.1 kDa). (c) DSC thermograms of P2VP (red) and P2VPNO.

### Small-angle X-ray Scattering (SAXS)

Figure 2(a) shows the background-corrected SAXS curves of P2VPNO (*M*_*n,cal*_ = 99.1 kDa)/D_2_O solutions at various P2VPNO concentrations: X05 (∼5 mg/mL), X10 (∼10 mg/mL), X20 (∼20 mg/mL), X30 (∼30 mg/mL), X40 (∼40 mg/mL) and X50 (∼50 mg/mL). D_2_O was used instead of H_2_O to ensure better consistency with the solutions for SANS experiments, where it served as the primary solvent component over H_2_O in the D_2_O/H_2_O mixtures. The SAXS curves were normalized by dividing each curve by the corresponding volume fractions (φ_*P*2*VPNO*_) listed in Table 1 for direct comparison between SAXS patterns, as shown in Figure 2(b). φ_*P*2*VPNO*_ were calculated based on the measured weights of P2VPNO and D_2_O used to prepare the solutions, as well as their mass densities obtained from the literature.^21,35^ Visual inspection of the normalized SAXS curves in Figure 2(b) reveals a subtle yet noticeable deviation in the SAXS intensity of the X10 solution (red circles), compared to the X05 solution (black circles) at the low**-** *q* (*q* ≤ 0.02 Å^–1^) region. From the X20 solution (blue circles) onward, these changes become more pronounced with increasing P2VPNO concentration. The observed deviations in the SAXS intensities are attributed to increasing interchain interactions among P2VPNO chains as the concentration increases. As concentration exceeds the overlap concentration, the P2VPNO chains no longer behave independently as in dilute condition. Instead, they begin to interpenetrate and interact with one another, and these interchain interactions influence the scattering pattern by introducing the contribution of the structure factor to the form factor. In SAXS, the observed scattered intensity, *I*(*q*), from solutions with interacting particles or polymer chains is typically expressed as the product of the form factor, *P*(*q*), and the structure factor, *S*(*q*), i.e., *I*(*q*)∼*P*(*q*)·*S*(*q*), where scattering vector *q* is given by, *q* = 4π·*sinθ*/λ, with θ is half of the scattering angle, and λ is being the wavelength of the X-ray or neutron beam.^36–38^ The form factor, *P*(*q*), describes the scattering from individual particles or polymer chains in isolation as observed in dilute solutions below the overlap concentration, while the structure factor, *S*(*q*), accounts for interactions between particles or chains which become more significant above the overlap concentration. If interchain interactions are absent, as observed under dilute conditions below overlap concentration, *S*(*q*) = 1, hence *I*(*q*) ∼ *P*(*q*). As interchain interactions increase at higher concentrations, *S*(*q*) deviates from unity and decreases toward zero at low-*q*, as shown later in Figure 5(a). At high-*q*, on the other hand, *S*(*q*) remains close to unity regardless of concentration. Consequently, as shown in Figure 2(b), the normalized SAXS intensity, *I*(*q*) ∼ *P*(*q*)·*S*(*q*), decreases at low-*q* with increasing concentration due to the contribution of *S*(*q*), while remaining largely unaffected at high-*q*. Below the overlap concentration, where *S*(*q*) = 1, the scattering is dominated by the single-chain form factor *P*(*q*). Since the single-chain conformation remains essentially unchanged regardless of concentration, SAXS curves normalized by volume fraction (or concentration) are expected to overlap, reflecting invariance of *P*(*q*). From this perspective, the small deviation of X10 curve at *q* ≤ 0.02 Å^–1^ from the X05 curve suggests that the P2VPNO concentration of X05 is close to the overlap concentration, where the scattering is dominated by *P*(*q*), with only minimal contribution from *S*(*q*), whereas X10 lies above the overlap concentration.

**Figure 2:**
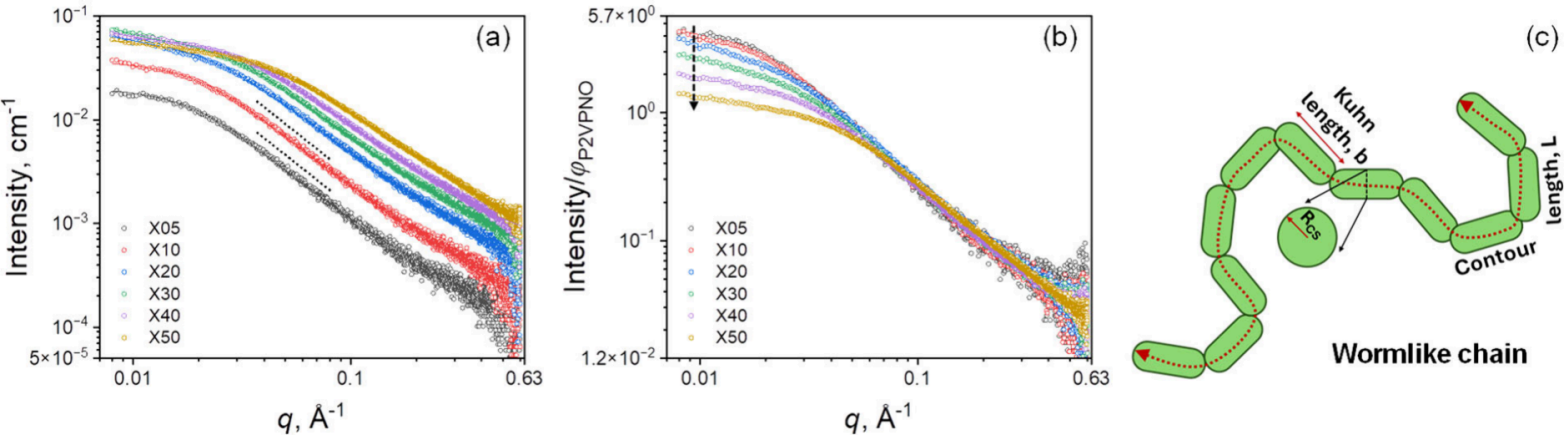
(a) SAXS of P2VPNO (*M*_*n,cal*_ = 99.1 kDa)/D_2_O solutions with varying P2VPNO volume fractions. The volume fractions of the solutions are listed in Table 1. In (a), the black dotted lines indicate *I­(q)* ∝ *q* ^–1.7^ scaling, which is a characteristic of self–avoiding chain (wormlike chain) conformation of semirigid polymers. (b) Volume fraction (φ_*P*2*VPNO*_)-normalized SAXS curves for each solution, where the absolute scale intensities were divided by the calculated volume fractions (φ_*P*2*VPNO*_’s). (c) Schematic representation of a wormlike chain with excluded volume effect, illustrating the contour length (*L*), Kuhn segmental length (*b*), and cross-sectional radius (*R*_*CS*_).

Under the hypothesis that P2VPNO assumes semirigid wormlike chain characteristics in D_2_O with excluded volume effect, we first analyzed the P2VPNO chain conformation by observing power–law behavior of the SAXS curves, see dotted lines in Figure 2(a). The *I (q)* ∝ *q* ^‑1.7^ scaling observed for solutions in the range of *q* = 0.04–0.09 Å^–1^ suggests that P2VPNO chains adopt a wormlike ‘swollen’ chain conformation with a mass fractal dimension (*D*_*f*_) of 1.7 as schematized in Figure 2(c).^38,39^

The small angle scattering, *I*(*q*) of wormlike chains with excluded volume interactions in solutions, characterized by contour length (*L*), Kuhn segment length (*b*) and cross-sectional radius (*R*_*CS*_), and effective excluded volume interaction parameter, *v*_*RPA*_, can be expressed as follows based on the Random Phase Approximation (RPA),^36,37,40–44^

1 $$I\left(q\right)=N_{0}\cdot {\left(\Delta \rho \right)}^{2}\cdot {\left\langle V\right\rangle }^{2}\cdot P\left(q\right)\cdot S\left(q\right)=N_{0}\cdot {\left(\Delta \rho \right)}^{2}\cdot {\left\langle V\right\rangle }^{2}\cdot \frac{P\left(q\right)}{1+v_{RPA}\cdot P\left(q\right)}$$

where *N*_0_ is number of chains in unit volume, i.e., number density. It is given by *N*_0_ = φ/⟨*V*⟩, where φ and ⟨*V*⟩ are volume fraction and ensemble average volume of wormlike chains, respectively.^45^ *Δρ* are X-ray or neutron scattering length density (*x*SLD or *n*SLD) contrast between solvent and polymer chains. The structure factor, *S*(*q*) = 1/(1+*v*_*RPA*_·*P*(*q*)) characterizes the interactions between P2VPNOs as a function of *q*. An important parameter within this framework is the effective excluded volume parameter, *v*_*RPA*_, which quantifies the magnitude of interactions between polymer chains and can explicitly be written in terms of *S*(*q*) at zero scattering angle (or at *q* = 0), *S*(0) as^44^

2 $$v_{RPA}=\frac{1}{S\left(0\right)}-1$$

In the absence of interchain interactions, as in dilute solutions, *S*(0) = 1 and therefore *v*_*RPA*_ = 0. Equation 1 then reduces to a simple single-chain form–factor representation of wormlike chain, *I*(*q*) = *N*_0_·(Δρ)^2^·⟨*V*⟩^2^·*P*(*q*). Here, *P*(*q*) is a form factor defined as,^37^

3 $$P\left(q\right)=P_{WLC}\left(q,L,b\right)\cdot P_{CS}\left(q,R_{CS}\right)$$

where *P*_*WLC*_ (*q,L,b*) is the form factor of an infinitely thin wormlike chain with excluded volume effect given by,^37,46^

4 $$P_{WLC}\left(q,L,b\right)=\{\begin{array}{c} P_{WLC,1}\left(q,L,b\right),qb<3.1\\ P_{WLC,2}\left(q,L,b\right),qb\geq 3.1 \end{array}$$

with

$$P_{WLC,1}\left(q,L,b\right)=\left(1-w\left({qR}_{g}\right)\right)\frac{2}{u_{1}^{2}}\left(e^{{-u}_{1}}+u_{1}-1\right)+f_{corr}\left(q\right)w\left({qR}_{g}\right)\left[\begin{array}{l} 1.22{\left({qR}_{g}\right)}^{-1/0.585}+0.4288{\left({qR}_{g}\right)}^{-2/0.585}\\ -1.651{\left({qR}_{g}\right)}^{-3/0.585} \end{array}\right]+\frac{C\left(n_{b}\right)}{n_{b}}\left[\frac{4}{15}+\frac{7}{15u_{2}}-\left(\frac{11}{15}+\frac{7}{15u_{2}}\right)e^{{-u}_{2}}\right]$$

where

$$w\left(x\right)=0.5\left(1+\tanh\frac{x-1.523}{0.1477}\right)$$

$$u_{1}=\frac{Lb}{6}\left(1-\frac{3}{2n_{b}}+\frac{3}{{2n}_{b}^{2}}-\frac{3}{{4n}_{b}^{3}}\left(1-e^{-2n_{b}}\right)\right)q^{2}$$

$$R_{g}^{2}={\left(\alpha \left(n_{b}\right)\right)}^{2}\frac{Lb}{6}$$

$$u_{2}={\left(\alpha \left(n_{b}\right)\right)}^{2}\frac{q^{2}Lb}{6}$$

$$\alpha \left(x\right)={\left(1+{\left(\frac{x}{3.12}\right)}^{2}+{\left(\frac{x}{8.67}\right)}^{3}\right)}^{0.176/6}$$

$$n_{b}=\frac{L}{b}$$

$$C\left(n_{b}\right)=\{\begin{array}{c} 3.06n_{b}^{-0.44},L>10b\\ 1,L\leq 10b \end{array}$$

$$f_{corr}\left(q\right)=\{\begin{array}{c} 1,\frac{dP_{WLC,1}}{dq}\leq 0\\ 0,\frac{dP_{WLC,2}}{dq}>0 \end{array}$$

and

$$P_{WLC,2}\left(q,L,b\right)=\frac{a_{1}\left(q,L,b\right)}{{\left(qb\right)}^{4.95}}+\frac{a_{2}\left(q,L,b\right)}{{\left(qb\right)}^{5.29}}+\frac{\pi }{qL}$$

Unlike linear polymers, where the cross-sectional radius is typically assumed to be infinitely small, PZs often possess bulky side groups, making their cross-sectional radii non–negligible. Consequently, the scattering function (form factor) of the cross-sectional radius of a semirigid rod, *P*_*CS*_(*q*) needs to be coupled to *P*_*WLC*_(*q,L,b*) to describe the overall form factor of wormlike chain as shown in Equation 3, where *P*_*CS*_(*q*) is given by^37^

5 $$P_{CS}\left(q,R_{CS}\right)={\left[\frac{2J_{1}\left(q,\left\langle R_{CS}\right\rangle \right)}{q,\left\langle R_{CS}\right\rangle }\right]}^{2}$$

where *J*_1_(*x*) and ⟨*R*_*CS*_⟩ denote the Bessel function of the first kind and ensemble average of *R*_*CS*_ over Schulz distribution. Also, we added a power law term (*A*/*q* ^*n*^) together with flat incoherent background term (*I*_*FB*_) to *I*(*q*) in Equation 1 to account for the potential presence of chain aggregation in the semidilute solutions, as well as low-*q*, high-*q* background and other unknown background scattering which yields,

6 $$I_{Fit}\left(q\right)=I\left(q\right)+\frac{A}{q^{n}}+I_{FB}$$

where, *I*_*Fit*_(*q*) is the final model to fit SAXS and SANS data, *A* and *n* are parameters in the power–law term.

Using the fitting model in Equation 6 and the related equations, the SAXS curves of X05, X10, X20, X30, X40, and X50 solutions were simultaneously fitted, with the summary of the important fitting parameters presented in Table 3. In the simultaneous fitting, we calculated the *x*SLDs of D_2_O (ρ_*D*2*O*_) and P2VPNO (ρ_*P*2*VPNO*_), where Δρ = ρ_*D*2*O*_ – ρ_*P*2*VPNO*_, and used these as fixed fitting parameters. The *x*SLDs calculated using the mass densities and atomic compositions of D_2_O and P2VPNO are 9.37·10^–6^ and 10.3·10^–6^ Å^–2^, respectively, as listed in Table 3.^18,33^ We assumed that *L* and *R*_*CS*_ for the P2VPNO (*M*_*n,cal*_ = 99.1 kDa) remain invariant regardless of varying concentrations. Therefore, these parameters were treated as shared fitting parameters common to all P2VPNO/D_2_O solutions with different P2VPNO volume fractions (φ_*P*2*VPNO*_). For the X05 solution, *v*_*RPA*_ was fixed at 0, as the solution was thought to be in the range of dilute regime near the overlap concentration with only negligible interchain interaction. In contrast, for the solutions with higher P2VPNO concentrations, *v*_*RPA*_ was treated as an independent fitting parameter. Additional independent fitting parameters for each solution included the *b*, *A*, *n*, and *I*_*FB*_. The fitted *L* and *R*_*CS*_ were 794 ± 14.9 Å, and 4.59 ± 0.174 Å, respectively. Fitted *R*_*CS*_ was corroborated with the lengths of the 2-vinylpyridine-*N*-oxide side group of P2VPNO, as obtained using Avogadro software,^47,48^ where the length was 4.32 Å. The *R*_*g*_’s of P2VPNO in each solution were calculated using the fitted *L*, *b* and *R*_*CS*_ and listed in Table 3. Here, *R*_*g*_ is given by, *R*_*g*_ = (*b*/2·*L*/3 + *R*_*CS*_^2^/2)^1/2^.^14^

**Table 3:** Important Fitting Parameters for SAXS Data for P2VPNO (*M*_*n,cal*_ = 99.1 kDa)/D_2_O Solutions Obtained Using Equation 6 and the Calculated Radii of Gyration (*R*_*g*_’s)

Parameters	X05	X10	X20	X30	X40	X50
Contour length, *L* (Å)	794 ± 14.9					
Kuhn length, *b* (Å)	28.0 ± 0.910	27.2 ± 0.583	25.0 ± 0.319	21.2 ± 0.210	17.9 ± 0.166	16.6 ± 0.188
Radius, *R*_*CS*_ (Å)	4.59 ± 0.174					
Excluded volume parameter, *v*_*RPA*_	0	0.230 ± 0.0233	0.784 ± 0.0253	0.891 ± 0.0267	1.62 ± 0.0372	3.10 ± 0.120
*x*SLD_P2VPNO_, (10^–6^·Å^–2^)	10.3					
*x*SLD_D2O_, (10^–6^·Å^–2^)	9.43					
*R*_*g*_ (Å)	60.9 ± 3.12	60.1 ± 2.41	59.3 ± 1.82	53.1 ± 1.49	48.1 ± 1.36	47.0 ± 1.41

Figure 3(a) and 3(b) shows the changes in *R*_*g*_ and *v*_*RPA*_ as a function of PZ volume fraction (φ = φ_*P*2*VPNO*_ or φ_*P*4*VPNO*_). It is seen that *R*_*g*_ decreases with increasing φ, while *v*_*RPA*_ increases. To determine whether or not this observation is general, additional SAXS measurements were performed on P2VPNO with higher molecular weight (*M*_*n,cal*_ = 175 kDa) than the P2VPNO of *M*_*n,cal*_ = 99.1 kDa and on P4VPNO (*M*_*n,cal*_ = 50.7 kDa), which has a lower molecular weight than the P2VPNOs, as shown in Figures 4(a) and 4(b), respectively. For high-molecular-weight P2VPNO (*M*_*n,P2VPNO*_ = 175 kDa), after volume fraction normalization, the SAXS profiles of Y03 (∼3 mg/mL) and Y05 solution (∼5 mg/mL) fell onto the same curve with negligible difference. From the Y10 solution (∼10 mg/mL) onward, changes become more pronounced (see dotted vertical arrow in Figure 4(a). The identical SAXS profiles of Y03 and Y05 imply that these solutions remained in the dilute regime below overlap concentration and thus exhibited the same P2VPNO chain conformation. In contrast, the deviations observed for Y10 reflect interchain interaction that becomes significant above the overlap concentration. The SAXS profiles were fitted using Equation 6, and the extracted values of *R*_*g*_ and *v*_*RPA*_ were incorporated into Figure 3(a) and 3(b). For high-molecular-weight P2VPNO, similar trends were observed as with the lower-molecular-weight P2VPNO: **R*_*g*_* decreased above the overlap threshold, while *v*_*RPA*_ increased. In contrast, for P4VPNO with lower molecular weight than P2VPNOs, no significant changes were observed in the SAXS curve pattern across the tested concentrations, and, consequently, no appreciable changes in *R*_*g*_. This suggests that the solutions, at concentrations ranging from 3 mg/mL to 40 mg/mL, all remained below the overlap concentration. It is well established that overlap concentration (c*) of polymers increases with decreasing molecular weight. In a θ–solvent,c* is estimated as $c^{*}=\frac{M_{w}}{(4/3)\pi R_{g}^{3}N_{av}}$ ,^49^ where *N*_*av*_ being the Avogadro number.

**Figure 3:**
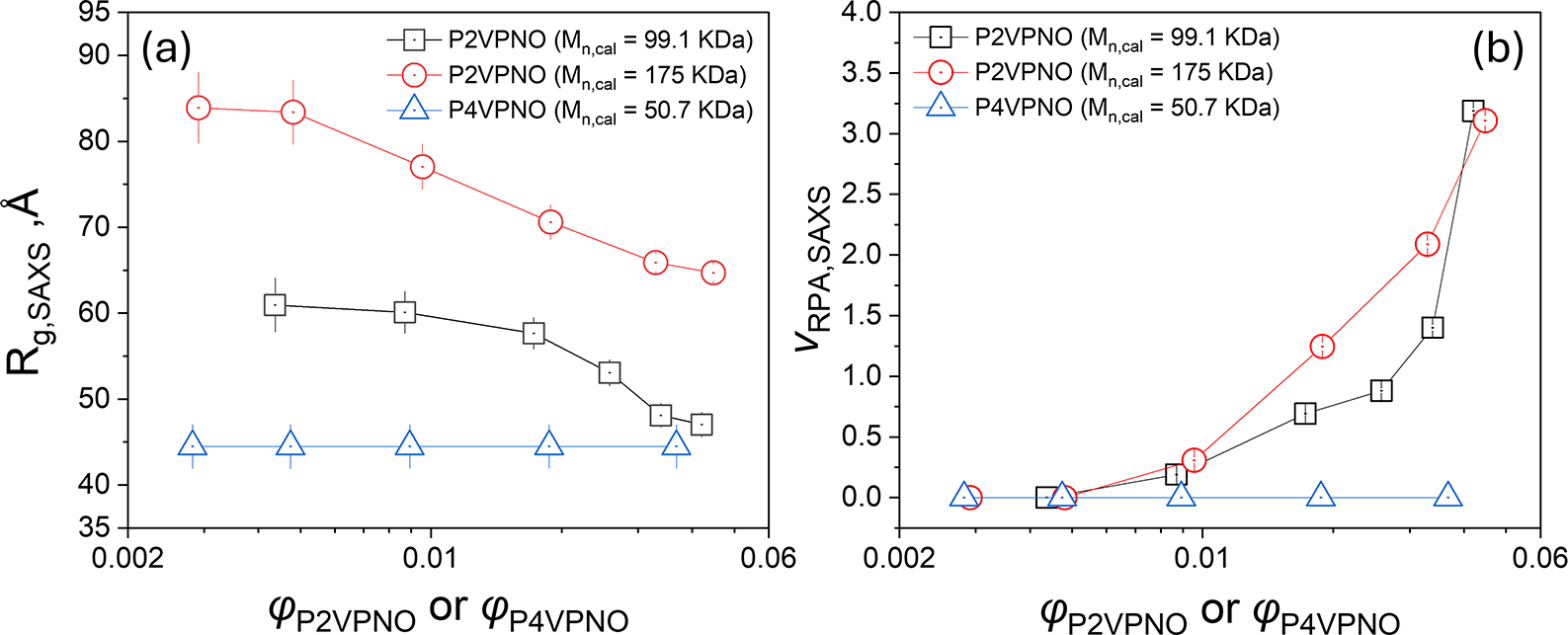
(a) *R*_*g*_ and (b) *v*_*RPA*_ as a function of φ (= φ_*P*2*VPNO*_ or φ_*P*4*VPNO*)_. *R*_*g*_ was calculated from the fit parameters, L, b and *R*_*CS*_ using *R*_*g*_ = (*b*/2·*L*/3 + *R*_*CS*_^2^/2)^1/2^.

**Figure 4:**
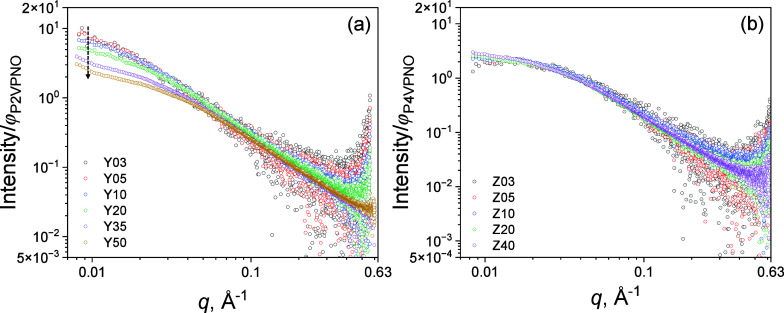
(a) Volume fraction-normalized SAXS curves for P2VPNO (*M*_*n,cal*_ = 175 kDa) and P4VPNO (*M_n,cal_* = 50.7 kDa) solutions, where the absolute scale intensities were divided by the calculated volume fractions.

In dilute solutions below the overlap concentration, polymer chains are well separated, and interchain interactions are negligible. Under these conditions, *b* and so *R*_*g*_ are primarily governed by intramolecular excluded volume effects within isolated chains and polymer–solvent interactions. As the concentration exceeds the overlap threshold, chains begin to interpenetrate, leading to the screening of intramolecular excluded volume interactions that stabilize extended conformations of chains at dilute concentrations, and the swelling of the chains is reduced.^14,15^ Consequently, the chains adopt more compact conformations, resulting in reduced *b* and, hence, in reduced *R*_*g*_. The observed concentration–induced collapse is consistent with de Gennes’ scaling theory for neutral linear polymer chains^14^ and is supported by dissipative particle dynamics (DPD) simulations,^15^ which show a systematic reduction in excluded volume effects with increasing polymer volume fraction:^14,15^

7 $$\frac{R_{g}}{R_{g,0}}∼{\left(\frac{\varphi }{\varphi^{*}}\right)}^{-\left(2f-1\right)/\left(6f-2\right)}$$

where *R*_*g,*0_ is the radius of gyration in the dilute limit, φ* is the overlap volume fraction and *f* is Flory exponent governing the mass scaling of a flexible polymer chain in an athermal (“good”) solvent.

The concentration-dependency of *v*_*RPA*_ shown in Figure 3(b) can be explained within the following framework: Thermodynamically, the inverse of *S*_*RPA*_(0) in Equation 2 $\left(v_{RPA}=\frac{1}{S_{RPA}\left(0\right)}-1\right)$ is related to the derivative of osmotic pressure, Π with respect to solute concentration, *c*: $\frac{1}{S_{RPA}\left(0\right)}=\frac{1}{k_{B}T}\cdot \frac{\partial \Pi }{\partial c}$ ,^43^ where *k*_*B*_ is the Boltzmann constant, *T* is the temperature. This expression along with Equation 2 indicates that *v*_*RPA*_ is directly related to Π, which reflects interchain interactions in the solution. In polymer solutions, Π can be expanded in a virial series: $\Pi =\frac{k_{B}T}{M}c+A_{2}c^{2}+A_{3}c^{3}+...$ , where *M* is the molecular weight of the polymer, *A*_2_, *A*_3_, etc., are the second, third, and higher-order virial coefficients representing the strength of two-body, three-body, and higher-order interactions, respectively.^14^ The dependence of Π on *c* illustrates that *v*_*RPA*_ is also concentration-dependent, i.e.; *v*_*RPA*_ ∝ *c*. On the other hand, the positive values of *v*_*RPA*_ in Figure 3(b) indicate that the interactions between wormlike chains are repulsive, with the magnitude of repulsion increasing as the concentration of polymer chain increases.^36,40^

Figures 5(a) and 5(b) display *S*(*q*) and *P*(*q*) derived from the fitted SAXS curves for the lower-molecular-weight P2VPNO (*M*_*n,cal*_ = 99.1 kDa)/D_2_O solutions in Figure 2(a). *S*(*q*) shown in Figure 5(a) reveal that the low-*q* scattered intensity decreases below unity progressively with increasing P2VPNO volume fraction (vertical arrow), indicating an increase in excluded volume interactions. At high-*q*, on the other hand, *S*(*q*) remains largely unchanged from unity regardless of volume interactions. In *P*(*q*) profiles shown in Figure 5(b), the power-law region in the intermediate-*q* region (0.03 ≤ *q* ≤ 0.1Å^–1^) shifts toward higher-*q* with increasing concentration, as indicated by the dotted horizontal arrow, consistent with a reduction in *b* shown in the inset of Figure 5(b).

**Figure 5:**
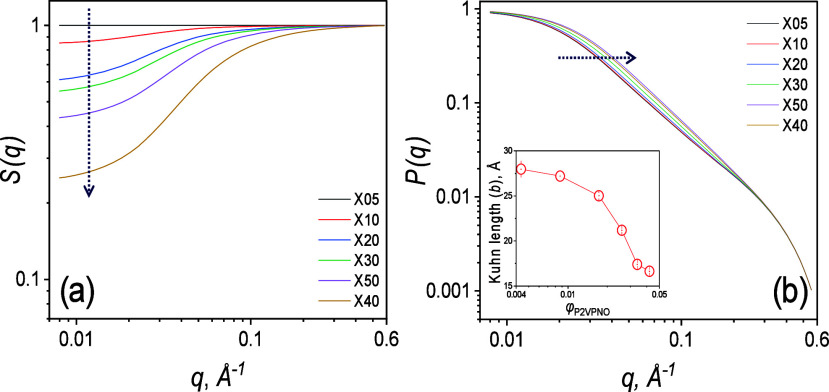
(a) SAXS structure factors, *S*(*q*) and (b) form factors, *P*(*q*) of P2VPNO/D_2_O solutions extracted from SAXS model-fits.

### Small-Angle Neutron Scattering (SANS)

To investigate the effect of salinity and temperature on the chain conformation, we chose P2VPNO with *M*_*n,cal*_ of 99.1 kDa at a polymer concentration of approximately 20 mg/mL. This concentration was chosen on the basis of the concentration-dependent SAXS results, which indicated that P2VPNO solutions at ∼20 mg/mL lie within the semidilute regime, i.e., above the overlap concentration. Accordingly, the SANS model-fit was performed using Equation 6 with nonzero *v*_*RPA*_. Figure 6(a) shows global SANS model-fits to the contrast-varied data sets of P2VPNO aqueous solutions (N1–N4) measured at 20 °C. Contrast (Δρ) variation was achieved by systematically adjusting the solvent D_2_O:H_2_O ratio: 100:0 (N1), ∼95:5 (N2), ∼90:10 (N3), and ∼85:15 (N4) % w/w, see Table 2. All solutions were prepared at nearly the same P2VPNO concentration (∼20 mg/mL), ensuring that polymer concentration—and thus chain conformation—remained consistent across the different D_2_O:H_2_O ratios. The only difference among the solutions was the *n*SLD contrast (Δρ) between polymer and solvent, yielding four distinct SANS curves (see Figure 6 (a)). Because the P2VPNO chains in all four D_2_O/H_2_O solvents adopt the same conformation, the chain conformation and hence the key structural parameters—*L*, *b*, *R*_*CS*_ and *v*_*RPA*_—should remain constant regardless of the D_2_O:H_2_O ratio and thus independent of the different SANS curves. By globally fitting all four SANS curves simultaneously with the key parameters constrained to be identical, we obtained shared structural values common to all four solutions with higher confidence than a single model-fit for single SANS data without contrast variation, thereby enhancing both the robustness and accuracy of the results.

**Figure 6:**
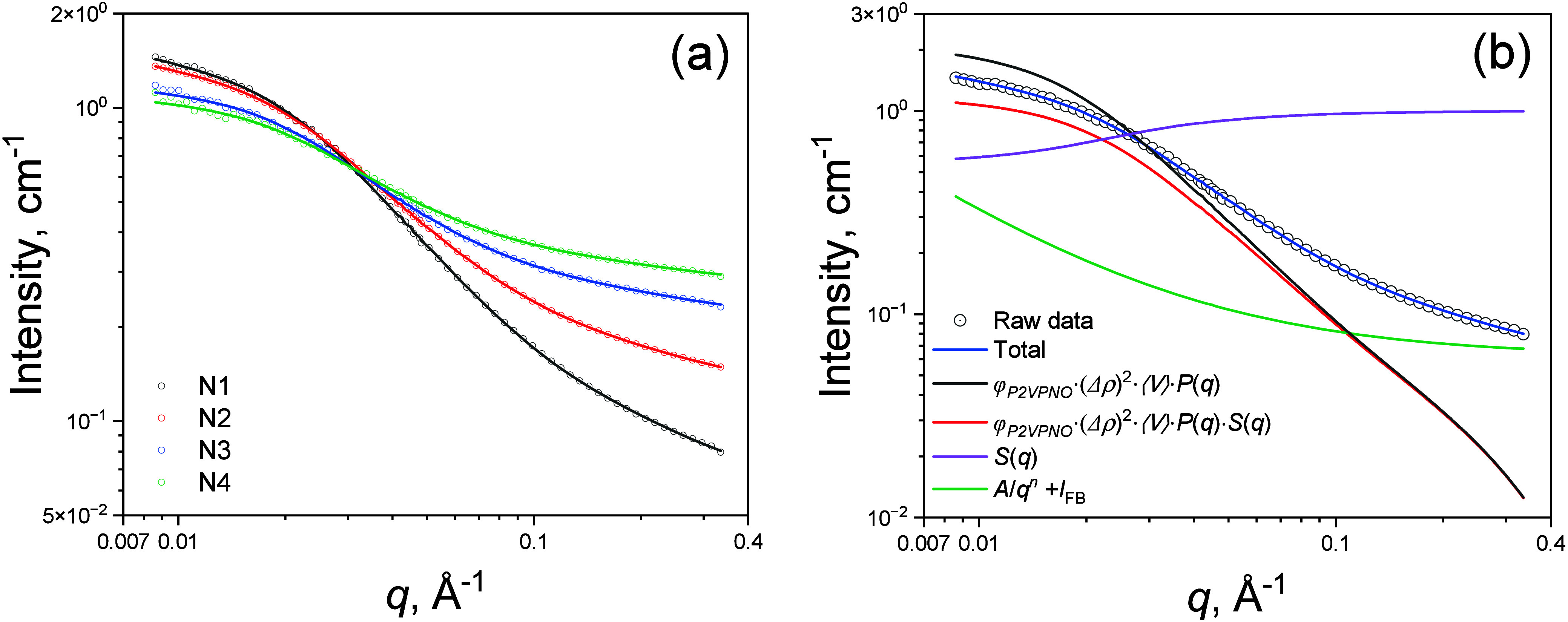
(a) SANS curves for N1, N2, N3, and N4 solutions measured at 20 °C. Solid lines represent global fits to the data using equation 6. Background scattering from an empty quartz cell (2 mm path length) was also measured and subtracted from the solution data. (b) SANS curve of the N1 solution at 20 °C (black circles) with the corresponding global fit result using Equation 6 (blue solid line). (b) also shows extracted φ_*P*2*VPNO*_·(Δρ)^2^·⟨*V*⟩·*P*(*q*), φ_*P*2*VPNO*_·(Δρ)^2^·⟨*V*⟩·*P*(*q*)·*S*(*q*), *S*(*q*), and *A*/*q* ^*n*^ + *I*_*FB*_.

To further increase reliability, measurable physical quantities before SANS model-fitting such as the *n*SLD contrast, i.e., (Δρ)^2^ and φ_*P*2*VPNO*_’s, were calculated and set as fixed fitting parameters. This reduces the number of free fitting parameters, minimizes overfitting and ensures that the fitted values remain within physically meaningful ranges. For this constrained fitting, the volume fractions of D_2_O and H_2_O in each solvent mixture were calculated using the measured weights and mass densities of D_2_O (1.10 g/cm^3^) and H_2_O (0.997 g/cm^3^).^33^ Using these volume fractions, the solvent *n*SLDs (100:0, ∼95:∼5, ∼90:∼10, and ∼85:∼15 wt % D_2_O:H_2_O) of solvent mixtures were determined to be 6.38 × 10^–6^, 6.02 × 10^–6^, 5.68 × 10^–6^, and 5.34 × 10^–6^ Å^–2^, respectively, as summarized in Table 2. The *n*SLD of P2VPNO, calculated using its mass density (∼1.14 g/cm^3^),^18^ was determined to be 2.01 × 10^–6^ Å^–2^. In the model-fits, *L* (∼794 Å) and *R*_*CS*_ (∼4.59 Å) obtained from SAXS analysis were also adopted and kept fixed, while *b* and *v*_*RPA*_ constrained to be identical in fitting all four SANS data sets. The global free fit parameters common to all N1, N2, N3, and N4 solutions at 20 °C were *b* = 30.1 ± 0.18 Å and *v*_*RPA*_ = 0.527 ± 0.011 as listed in Table 4. Using *L* (∼794 Å) and *R*_*CS*_ (∼4.59 Å) obtained from SAXS analysis, together with *b* (*∼*30.1Å) from SANS analysis, *R*_*g*_ at 20 °C was ∼63.2 Å.

**Table 4:** Important Fitting Parameters for SANS data of N1, N2, N3, and N4 Measured at 20 °C

Parameters	N1	N2	N3	N4
Volume fraction, φ_P2VPNO_	0.0192	0.0192	0.0191	0.0189
Contour Length, *L* (Å)	794 ± 14.9			
Kuhn Length, *b* (Å)	30.1 ± 0.18			
Radius, *R*_*CS*_ (Å)	4.59 ± 0.174			
Excluded volume parameter, *v*_*RPA*_	0.527 ± 0.0107			
*n*SLD_P2VPNO_ (10^–6^·Å^–2^)	2.01			
*n*SLD_Solvent_(10^–6^·Å^–2^)	6.34	5.98	5.65	5.31
*R*_*g*_ (Å)	63.2 ± 0.35			

To investigate the effect of temperature on the P2VPNO chain conformations, we conducted additional contrast-variation SANS measurements at 40 °C, 55 °C, and 60 °C on the same N1, N2, N3, and N4 solutions. From each global SANS model-fit, the extracted *R*_*g*_ and *v*_*RPA*_ are co-plotted in Figure 7(a) and 7(b) for comparison with those obtained at 20 °C. Furthermore, to study the combined effect of KBr concentration and temperature on chain conformation, particularly focusing on the anti-polyelectrolyte effect, we prepared aqueous solutions containing 1:1 (N5, N6, N7 and N8) and 1:10 (N9, N10, N11 and N12) molar ratios of [VPNO]:[KBr] by dissolving in four distinct D_2_O/H_2_O mixtures with ratios of 100:0, ∼95:5, ∼90:10, and ∼85:15% w/w. We then conducted contrast-variation SANS measurements on the prepared solutions at 20, 40, 55, and 60 °C. To determine the *n*SLDs of solvents containing different molar ratios of KBr, we calculated the volume fractions (φ_*P*2*VPNO*_’s) of D_2_O, H_2_O, and KBr using their weights and mass densities–1.10 g/cm^3^ for D_2_O, 0.997 g/cm^3^ for H_2_O, and 2.75 g/cm^3^ for KBr. The resulting *n*SLDs of each KBr/D_2_O/H_2_O solvent are summarized in Table 2, and these *n*SLDs were held fixed during the global fits of each SANS data set. Also, the φ_*P*2*VPNO*_’s of the P2VPNO in each P2VPNO/KBr/D_2_O/H_2_O solution were calculated and kept fixed for the constrained global fitting of the SANS data sets. The resulting *R*_*g*_ and *v*_*RPA*_ as a function of temperature and salt concentration are added in Figures 7(a) and 7(b), respectively, and summarized in Table 5. Here, *R*_*g*_ was calculated from *b*, *L* and *R*_*CS*_, according to *R*_*g*_ = (*b*/2·*L*/3 + *R*_*CS*_^2^/2)^1/2^. Since *L* and *R*_*CS*_ remain constant for this P2VPNO (*M*_*n,cal*_ = 99.1 kDa), the variation in *R*_*g*_ is governed solely by *b*, which quantifies chain rigidity. Notably, *b* decreases with increasing temperature, as described by the well–known equation, $b\left(T\right)=\frac{2B}{k_{B}T}$ , where *B* is the bending modulus; thus, chain becomes more flexible at higher temperature. The reduction in *b* and, consequently, *R*_*g*_ shown in Figure 7(a) is primarily due to thermal fluctuations, which increase the flexibility of the polymer chains by lowering the energy barriers for bond rotation and segmental motion.^50,51^ As temperature increases, promoted vibrational and rotational freedom within the P2VPNO molecular structure reduces chain rigidity, leading to a decrease in *b*, which results in a reduction of *R*_*g*_.^46^ The temperature dependent reduction of *v*_*RPA*_, on the other hand, can be explained as follows. As *b* decreases with increasing temperature, P2VPNO adopts a more compact chain conformation, leading to a smaller *R*_*g*_ and reduced excluded volume per P2VPNO chain. Thus, even at constant P2VPNO chain concentration, the reduced excluded volume lowers the degree of chain overlap and consequently reduces the excluded volume interaction, *v*_*RPA*_ between P2VPNO chains, as shown in Figure 7(b).^47^ Figure 8 shows a schematic illustrating the temperature dependence of *R*_*g*_, degree of chain overlap and *v*_*RPA*_ for P2VPNO chains in semidilute solutions. An increase in temperature leads to a reduction in *R*_*g*_, resulting in the degree of chain interpenetration (overlap) thereby reducing *v*_*RPA*_ between neighboring P2VPNO chains. As is common for PZs, P2VPNO also exhibits an anti-polyelectrolyte effect under varying salinity conditions, whereby added salt increases *b* and consequently enlarges *R*_*g*_, while increasing *v*_*RPA*_ as indicated by the vertical arrows in Figure 7(a) and Figure 7(b). An especially interesting observation is that, at elevated temperatures, chain shrinkage occurs due to increased chain flexibility; nevertheless, the salinity effect persists, sustaining the salt-induced chain expansion.

**Table 5:** Radii of Gyration (*R*_*g*_’s) and Excluded Volume Parameters (*v*_*RPA*_’s) Obtained from the Constrained Co-refinement of SANS Data

	[VPNO]:[KBr]1:0 (N1, N2, N3, and N4)		[VPNO]:[KBr]1:1 (N5, N6, N7, and N8)		[VPNO]:[KBr]1:10 (N9, N10, N11, and N12)	
Temperature, °C	*R*_*g*_, Å	*v*_*RPA*_	*R*_*g*_, Å	*v*_*RPA*_	*R*_*g*_, Å	*v*_*RPA*_
20	63.2 ± 0.35	0.527 ± 0.0107	64.7 ± 0.37	0.674 ± 0.0161	67.4 ± 0.509	0.892 ± 0.0235
40	60.8 ± 0.32	0.477 ± 0.0108	62.2 ± 0.38	0.558 ± 0.0144	64.8 ± 0.44	0.699 ± 0.0196
55	59.9 ± 0.30	0.456 ± 0.0105	60.2 ± 0.38	0.526 ± 0.0134	62.6 ± 0.40	0.574 ± 0.0170
60	59.0 ± 0.37	0.447 ± 0.0134	59.8 ± 0.38	0.498 ± 0.0156	61.7 ± 0.48	0.537 ± 0.0200

**Figure 7:**
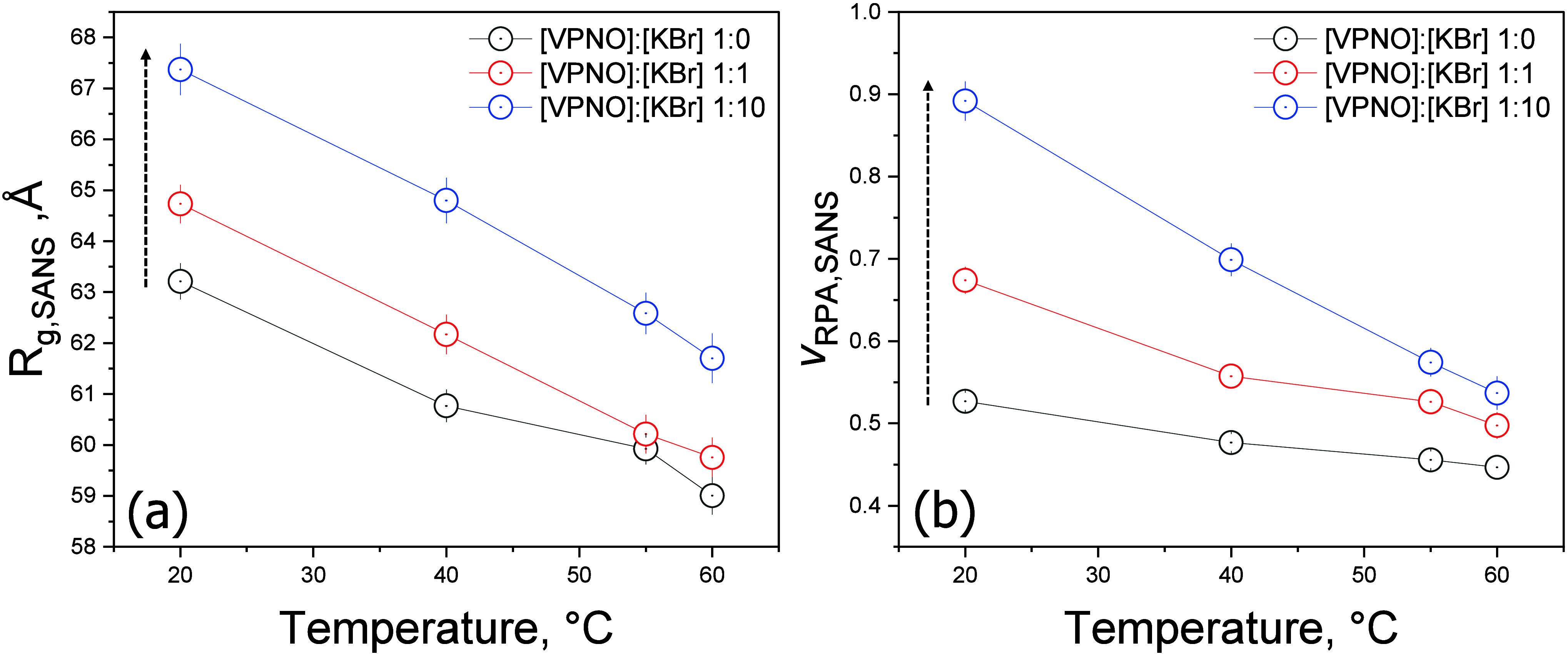
(a) *R*_*g*_ and (b) *v*_*RPA*_ as a function of temperature and the molar ratio of KBr. Here, *R*_*g*_ = (*b*/2·*L*/3 + *R*_*CS*_^2^/2)^1/2^.

**Figure 8:**
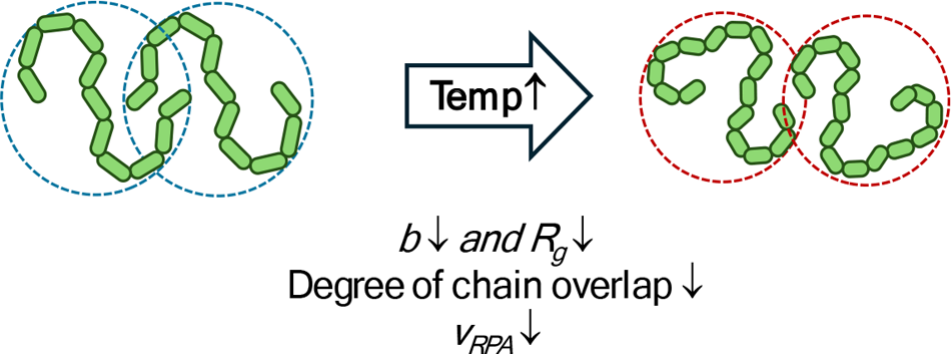
Schematic illustration of the temperature dependence of *b* and *R*_*g*_, chain overlap and *v*_*RPA*_ for P2VPNO in semidilute solutions.

In PZs, *R*_*g*_ typically increases upon salt addition because added counterions screen the intramolecular electrostatic attractions between the oppositely charged zwitterionic segments. The added cations and anions interact with the oppositely charged ions on the zwitterionic groups along the polymer chain, weakening dipolar interactions and promoting a transition toward a more expanded chain conformation. For instance, PZs, such as PSBMA^8,16,52,53^ and PAEDAPS,^6^ exhibit strong salt-dependent changes in chain conformation. When salt is introduced, salt ions screen intramolecular interactions. This screening allows polymer chains to adopt more open conformations, effectively increasing *R*_*g*_. In this case, increasing the salt content in the PZ aqueous solution is expected to continue expanding *R*_*g*_ until all residual intramolecular associations are fully disrupted by added salt and saturated. This salt-driven chain expansion ultimately depends on the intrinsic dipole moment of the zwitterionic repeating units, which governs the balance between intramolecular dipolar interactions and polymer–water interactions, both of which determine the chain conformation of PZs in aqueous solutions. A higher dipole moment strengthens intrachain dipolar attractions, favoring intramolecular association, whereas stronger polymer–water interactions promote chain expansion (hydration). In this study, P2VPNO chains predominantly adopt a pre–expanded, wormlike conformation in salt-free solution, driven by hydration of the zwitterionic N^+^–O^–^ groups in the pyridine-*N*-oxide repeat units. Salt addition further increases *R*_*g*_, and this expansion persists at elevated temperatures (Figure 7(a)). A distinctive feature of P2VPNO is its unusually short one-bond-long charge separation length, which reduces its dipole moment compared to PSBMA and PAEDAPS. As a result, intrachain associations are weaker, favoring expanded conformations even under salt-free conditions. In contrast, PSBMA, with its larger dipole moment, forms compact Gaussian coil–like conformations in the absence of salt, but expands upon salt addition via the anti-polyelectrolyte effect.^8,53^ Overall, molecular parameters such as dipole length, and hydration capacity dictate the balance of intramolecular versus polymer–solvent interactions, governing chain conformation and salt responsiveness in PZs. Understanding this interplay provides a foundation for tailoring PZ properties, including solubility, mechanical strength, and environmental responsiveness.

## Conclusions

This study examined the conformational behavior of P2VPNO as a function of polymer concentration (SAXS), and salinity and temperature (SANS). In salt-free aqueous solutions, P2VPNO chains predominantly adopted an expanded, wormlike conformation with excluded volume effects. This behavior contrasts with PSBMA, which adopts a more compact, Gaussian–like conformation under salt-free conditions due to its longer dipole length and stronger dipole moment. With increasing P2VPNO concentration, interchain interpenetration becomes more significant, leading to an increase in *v*_*RPA*_. At the same time, the interpenetrated chains screen the intrachain excluded–volume interactions, causing a contraction of *R*_*g*_. As in many PZs, added salt in P2VPNO solutions screens intrachain dipolar attractions, increases *b*, and thereby expands the polymer chain via the anti-polyelectrolyte effect. At elevated temperatures, greater thermal motion reduces chain rigidity, decreasing both *R*_*g*_ and *v*_*RPA*_; nevertheless, salinity continues to induce chain expansion relative to salt-free conditions, thereby sustaining the anti-polyelectrolyte response. Overall, these results provide important new insights into the solution physics of polyzwitterions.

## Supplementary Material



## Notes

Supplementary experimental data including solution volume fractions; polymer ^1^H-NMR spectra; polymer SEC chromatograms (PDF)
