# Supplementary material for: Effects of Concentration, Salinity and Temperature on the Conformations of Zwitterionic Poly(2-Vinylpyridine‑N‑Oxide) Chains in Semidilute Solutions Probed by Small-Angle X‑Ray and Neutron Scattering
Source: Macromolecules. 2026 Mar 27;59(7):4015–27. doi: 10.1021/acs.macromol.5c02665 (PMC13085816; doi:10.1021/acs.macromol.5c02665)
Supplement: Supplementary file 1 [file ma5c02665_si_001.pdf]

Supporting Information

Effects of Concentration, Salinity and Temperature on the Conformations of Zwitterionic Poly(2–Vinylpyridine–*N*–Oxide) Chains in Semidilute Solutions Probed by Small–Angle X–Ray and Neutron Scattering[†]

Polyxeni P. Angelopoulou[1,2], Jong K. Keum[2,3,*], Pei-Chi Chen[4], Guang–Rong Huang[4,5], Changwoo Do[3], Logan T. Kearney[1], Jan Michael Carrillo[2], Yangyang Wang[2], Jack F. Douglas[6], Panagiotis Christakopoulos[2], Rajeev Kumar[2], Georgios Sakellariou[7], Amit K. Naskar[1], Kunlun Hong[2,*]

[1]Chemical Sciences Division, Oak Ridge National Laboratory, Oak Ridge, TN 37830, USA

[2]Center for Nanophase Materials Sciences, Oak Ridge National Laboratory, Oak Ridge, TN 37830, USA

[3]Neutron Scattering Division, Oak Ridge National Laboratory, Oak Ridge, TN 37830, USA

[4]Department of Engineering and System Science, National Tsing Hua University, Hsinchu 300044, Taiwan.

[5]Physics Division, National Center for Theoretical Sciences, Taipei 10617, Taiwan

[6]NIST Fellow Emeritus, National Institute of Standards and Technology, Gaithersburg, Maryland 20899, USA

[7]Department of Chemistry, National and Kapodistrian University of Athens, Athens, 15771, Greece.

Email: keumjk@ornl.gov, kunlun7@gmail.com

[†]This manuscript was authored by UT–Battelle, LLC under Contract No. DE–AC05–00OR22725 with the U.S. Department of Energy. The United States Government retains and the publisher, by accepting the article for publication, acknowledges that the United States Government retains a non–exclusive, paid–up, irrevocable, world–wide license to publish or reproduce the published form of this manuscript, or allow others to do so, for United States Government purposes. The Department of Energy will provide public access to these results of federally sponsored research in accordance with the DOE Public Access Plan (http://energy.gov/downloads/doe–public–access–plan).

Table S1. P2VPNO/D_2O solutions with P2VPNO of $M_{n,cal}$ = 175 kDa at different volume fractions (φ_{P2VPNO}), prepared for SAXS measurements.

**Solution ID**	**Volume fraction, φ_{P2VPNO}**
Y03	0.0029
Y05	0.0048
Y10	0.0096
Y20	0.0189
Y35	0.0330
Y50	0.0448

Table S2. P4VPNO/D_2O solutions prepared for SAXS measurements with P4VPNO of $M_{n,cal}$ = 50.7 kDa at different volume fractions (φ_{P4VPNO}), prepared for SAXS measurements.

**Solution ID**	**Volume fraction, φ_{P4VPNO}**
Z03	0.0028
Z05	0.0047
Z10	0.0089
Z20	0.0187
Z40	0.0368

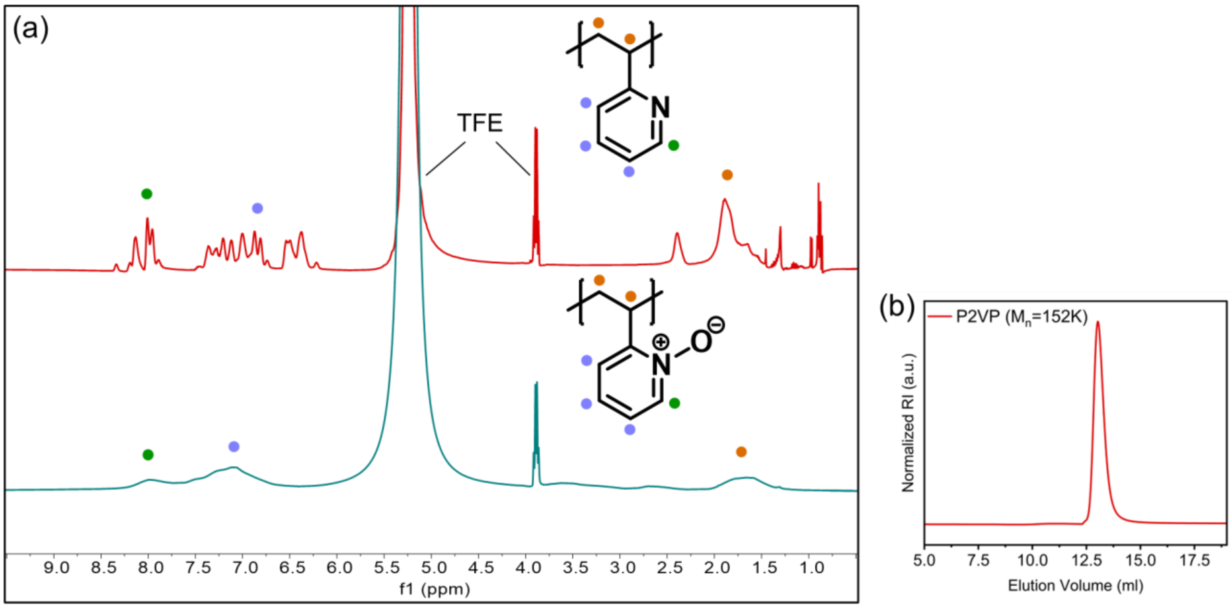

Figure S1. (a) ^{1}H-NMR spectra of P2VP (M_n = 152 kDa) and P2VPNO ($M_{n,cal}$ = 175 kDa) in TFE–d_2. (b) Size exclusion chromatography (SEC) trace in THF of parent P2VP (M_n = 152 kDa) before oxidation towards P2VPNO.

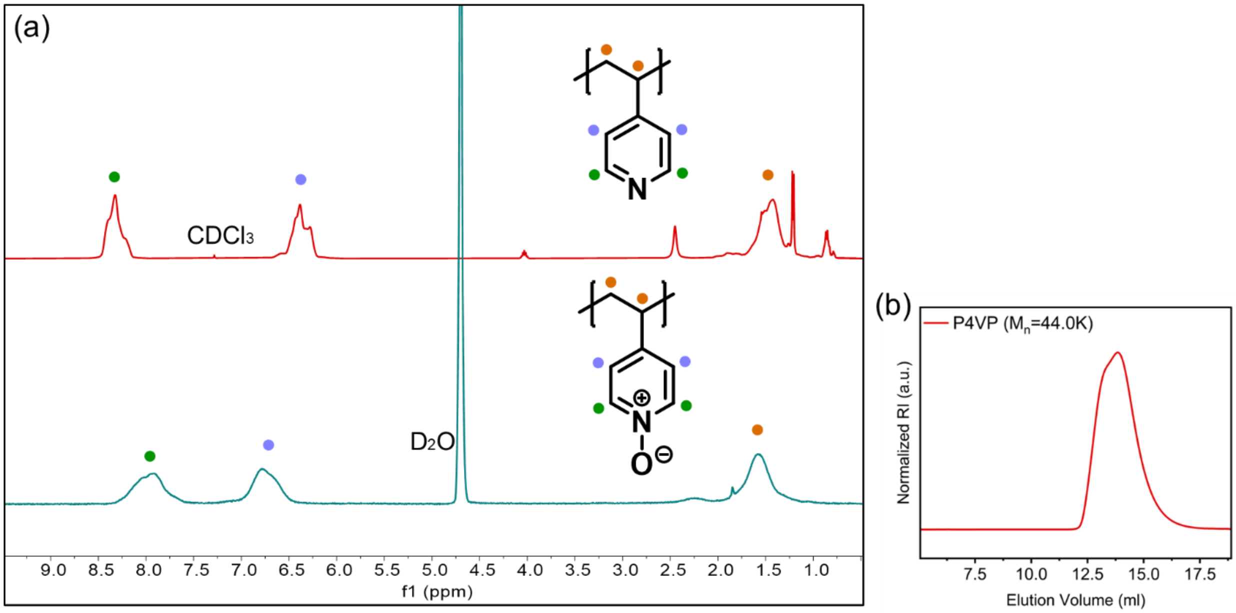

Figure S2. (a) ^{1}H-NMR spectra of P4VP (M_n = 44.0 kDa) and P4VPNO ($M_{n,cal}$ = 50.7 kDa) in $CDCl_3$ and D_2O, respectively. (b) Size exclusion chromatography (SEC) trace in DMF of parent P4VP (M_n = 44.0 kDa) before oxidation towards P4VPNO.
